# Systematic assignment of thermodynamic constraints in metabolic network models

**DOI:** 10.1186/1471-2105-7-512

**Published:** 2006-11-23

**Authors:** Anne Kümmel, Sven Panke, Matthias Heinemann

**Affiliations:** 1 Institute of Molecular Systems Biology, ETH Zurich, Wolfgang-Pauli-Str. 16, 8093 Zurich, Switzerland; 2 Bioprocess Laboratory, Institute of Process Engineering, ETH Zurich, Universitätsstr. 6, 8092 Zurich, Switzerland

## Abstract

**Background:**

The availability of genome sequences for many organisms enabled the reconstruction of several genome-scale metabolic network models. Currently, significant efforts are put into the automated reconstruction of such models. For this, several computational tools have been developed that particularly assist in identifying and compiling the organism-specific lists of metabolic reactions. In contrast, the last step of the model reconstruction process, which is the definition of the thermodynamic constraints in terms of reaction directionalities, still needs to be done manually. No computational method exists that allows for an automated and systematic assignment of reaction directions in genome-scale models.

**Results:**

We present an algorithm that – based on thermodynamics, network topology and heuristic rules – automatically assigns reaction directions in metabolic models such that the reaction network is thermodynamically feasible with respect to the production of energy equivalents. It first exploits all available experimentally derived Gibbs energies of formation to identify irreversible reactions. As these thermodynamic data are not available for all metabolites, in a next step, further reaction directions are assigned on the basis of network topology considerations and thermodynamics-based heuristic rules. Briefly, the algorithm identifies reaction subsets from the metabolic network that are able to convert low-energy co-substrates into their high-energy counterparts and thus net produce energy. Our algorithm aims at disabling such thermodynamically infeasible cyclic operation of reaction subnetworks by assigning reaction directions based on a set of thermodynamics-derived heuristic rules. We demonstrate our algorithm on a genome-scale metabolic model of *E. coli*. The introduced systematic direction assignment yielded 130 irreversible reactions (out of 920 total reactions), which corresponds to about 70% of all irreversible reactions that are required to disable thermodynamically infeasible energy production.

**Conclusion:**

Although not being fully comprehensive, our algorithm for systematic reaction direction assignment could define a significant number of irreversible reactions automatically with low computational effort. We envision that the presented algorithm is a valuable part of a computational framework that assists the automated reconstruction of genome-scale metabolic models.

## Background

Nowadays, high-throughput experimental omics techniques are being developed and are generating large-scale data sets and information bases that can hardly be intuitively understood. Models that enable mathematical analysis and simulation are essential to benefit from the knowledge that is contained in these data sets. Consequently, the importance of models increases along with the advances in experimental technologies.

One class of models that has particularily proven to be useful for the analysis of omics data is the class of stoichiometric metabolic models [1,2]. Several such models – today typically available on genome-scale – were reconstructed for various organisms (e.g. [3-5]) and are used as tools in systems biology [6,7] and metabolic engineering [8,9]. Genome-scale stoichiometric models are composed of the metabolic reactions' stoichiometry and assignments of the reactions' reversibility or irreversibility.

In the model reconstruction process – reviewed in [10] – typically first a preliminary organism-specific metabolic network is generated by drawing on information stored in sequence databases that link coding regions to cellular functions. In the next step, the sequence-derived network is completed with knowledge from biochemistry and physiology such that a stoichiometric network is derived that reflects the cell's metabolic capabilities. For the reconstruction of metabolic reaction networks and particularily for the identification of enzymes that lack genetic evidence, a series of computational tools exist [11-13].

In contrast, reaction directions are often assigned manually, or are adopted from other existing models or databases on metabolic pathways (e.g. KEGG). Direction assignment is not only laborious but also error-prone due to manual execution. Indeed, it was shown that the direction assignments of published genome-scale models contain inconsistencies i.e. reaction directions that contradict each other (M. Terzer and J. Stelling, *personal communication*). Error diagnostics in these cases is difficult as the underlying reasons for direction assignments are not provided in the currently available models.

Reaction directionalities are used frequently: First, they are required for analysis and simulation of metabolic phenomena by constraint-based modeling [2]. Second, the reactions' directionality is usually reported in maps on metabolic pathways within widely-used genomic databases such as KEGG or MetaCyc [14,15]. Third, the information on reactions' (ir)reversibility is essential for metabolic flux analysis [16].

In principle, all biochemical reactions are reversible: A reaction can proceed in either forward or backward direction depending on the actual Gibbs energy of reaction. The Gibbs energy of reaction is determined by the reactants' standard Gibbs energies of formation and their concentrations. A change in reactant concentrations, for example, can reverse a reaction's direction, if the respective Gibbs energy of reaction turns from a negative to a positive value. There are, however, so-called irreversible reactions that under physiological conditions only proceed in one direction, which means that the respective reactants' Gibbs energies of formation and concentrations are always such that only one direction is possible.

The natural approach to identify the irreversible reactions in stoichiometric models would draw on Gibbs energies of formation and physiological concentration ranges. However, experimentally determined Gibbs energies of formation are not available for all metabolites. As a workaround, a group contribution method was developed that computationally estimates Gibbs energies of formation for a large set of metabolites [17]. Using the values obtained with this method and taking into account its inherent significant uncertainties, a genome-scale thermodynamic analysis of *E. coli*'s metabolism showed that only five reactions (out of 873) could be classified as irreversible [18]. This very small number demonstrates that computationally estimated Gibbs energies of formation are too uncertain to be used to assign reaction directions.

An alternative approach to assign reaction directions is to apply the nonlinear constraint that arises from the fact that there must exist a thermodynamic driving force for any mass-balanced set of reaction fluxes in a reaction network [19]. For internal reaction cycles that result in no net conversion of metabolites the overall thermodynamic driving force is zero, i.e. the cyclic operation of these reactions is infeasible. Given the specification of the directions of a subset of network fluxes (e.g. by using information about the environmental conditions to specify the exchange of metabolites with the environment), it was shown to be possible to compute the feasible direction of some of the not preset fluxes based on the nonlinear thermodynamic constraints [20]. This *ab initio *calculation of the reaction directions is based on an NP-complete computation [20]. As a result, a computationally effortless algorithm for the assignment of reaction directions (thermodynamics-based linear constraints) in genome-scale networks does not exist today.

Here, we present a computational method that is intended to close this gap. In a first step, our method draws on experimentally determined thermodynamic data, i.e. Gibbs energies of formation, and physiological intracellular metabolite concentrations to assign as many reaction directions as possible. Next, in order to assign further reaction directions, we draw on network topology and heuristic rules that exploit the knowledge of biochemical energy equivalents such as ATP or NADH. An algorithm that is capable to apply this procedure to genome-scale stoichiometric models was developed and implemented in Matlab. The respective script is available from the authors on request.

## Results

**Figure 1:**
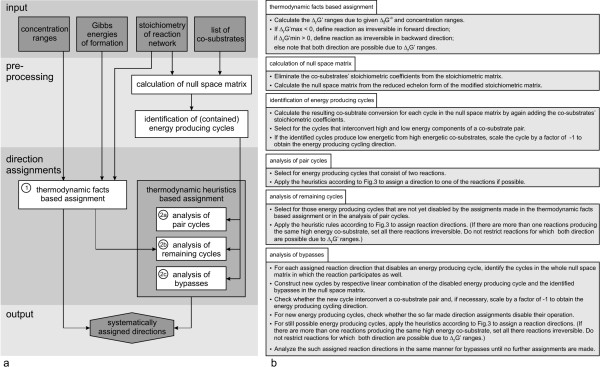
**Illustration of the algorithm for systematic assignment of reaction directions**. Panel *a *gives an overview over the direction assignment procedure. Each step (white boxes) is decribed in detail in panel *b*.

In the following, the algorithm (cf. overview in Fig. 1) is described in detail. Each step is illustrated by applying the procedure to the genome-scale reconstruction of *E. coli*'s metabolic network [4]. From this model, we only used the stoichiometric matrix but not the constraints that were placed on the reaction directions. In other words, we applied our algorithm to the metabolic network, in which initially all reactions were considered as reversible.

### Thermodynamic facts-based assignment

First, we aimed to assign as many directions as possible on solid thermodynamic grounds: A reaction can only proceed in direction of a negative Gibbs energy of reaction, Δ_*r*_*G*. The Gibbs energy of reaction depends on the reactants' *i *standard Gibbs energies of formation, Δ_*f*_Gi0
 MathType@MTEF@5@5@+=feaafiart1ev1aaatCvAUfKttLearuWrP9MDH5MBPbIqV92AaeXatLxBI9gBaebbnrfifHhDYfgasaacH8akY=wiFfYdH8Gipec8Eeeu0xXdbba9frFj0=OqFfea0dXdd9vqai=hGuQ8kuc9pgc9s8qqaq=dirpe0xb9q8qiLsFr0=vr0=vr0dc8meaabaqaciaacaGaaeqabaqabeGadaaakeaacqWGhbWrdaqhaaWcbaGaemyAaKgabaGaeGimaadaaaaa@3039@, their concentrations, *c*_*i*_, and the respective stoichiometric coefficients, *ν*_*i*_:

ΔrG=∑iνiΔfGi0+RTln⁡(∏iciνi).     (1)
 MathType@MTEF@5@5@+=feaafiart1ev1aaatCvAUfKttLearuWrP9MDH5MBPbIqV92AaeXatLxBI9gBaebbnrfifHhDYfgasaacH8akY=wiFfYdH8Gipec8Eeeu0xXdbba9frFj0=OqFfea0dXdd9vqai=hGuQ8kuc9pgc9s8qqaq=dirpe0xb9q8qiLsFr0=vr0=vr0dc8meaabaqaciaacaGaaeqabaqabeGadaaakeaacqqHuoardaWgaaWcbaGaemOCaihabeaakiabdEeahjabg2da9maaqafabaacciGae8xVd42aaSbaaSqaaiabdMgaPbqabaaabaGaemyAaKgabeqdcqGHris5aOGaeuiLdq0aaSbaaSqaaiabdAgaMbqabaGccqWGhbWrdaqhaaWcbaGaemyAaKgabaGaeGimaadaaOGaey4kaSIaemOuaiLaemivaqLagiiBaWMaeiOBa4MaeiikaGYaaebuaeaacqWGJbWydaqhaaWcbaGaemyAaKgabaGae8xVd42aaSbaaWqaaiabdMgaPbqabaaaaaWcbaGaemyAaKgabeqdcqGHpis1aOGaeiykaKIaeiOla4IaaCzcaiaaxMaadaqadaqaaiabigdaXaGaayjkaiaawMcaaaaa@54FF@

If it turned out in our analysis that with any physiologically reasonable reactant concentrations, the Gibbs energy of reaction for a given reaction was always negative, the reaction was defined as irreversible in the respective direction. For the Gibbs energy of formation, we employed experimentally derived values, which were available for 157 out of 761 metabolites present in the network (cf. Methods). Although a computational method can roughly estimate Δ_*f*_*G*^0^-values for many more molecules [17], we prefered to employ this limited set of thermodynamic data as only a very limited set of irreversible reactions could be assigned with computationally determined Δ_*f*_*G*^0^-values due to their inherent uncertainties (cf. Background and [18]).

Furthermore, Maskow and von Stockar have shown that only with Gibbs energies of formation, that are adjusted to physiological pH and ionic strength, e.g. a flux through glycolysis is thermodynamically feasible [21]. Thus, we considered physiological pH and ionic strength values (cf. Methods) by using the respectively transformed Gibbs energies of formation/reaction [22]. For simplicity, 'transformed Gibbs energies' will only be referred to as 'Gibbs energies' in the following.

Intracellular metabolite concentrations were also required to determine the actual Gibbs energies of reaction. These are widely unknown. As, moreover, any stoichiometric model is usually applied for a variety of growth conditions and even for mutant strains where different concentration levels can be conceived, we anyhow wanted to base our analysis on concentration ranges that cover a wide spectrum of conditions. Therefore, we here assumed broad physiological ranges for intracellular metabolite concentrations, which typically are in the order of *μM *to *mM *[23].

Employing a respective concentration range from 0.001 to 10 *mM *and by using the available set of experimental values for Gibbs energies of formation, ranges of Gibbs energies of reaction could be determined for 176 (out of 920) reactions in the model. In this set of ranges, we checked for allowed operational reaction directions: A positive (negative) direction was set if the range of Gibbs energy of reaction was exclusively negative (positive). With this approach, 43 reactions were defined as irreversible in the analyzed *E. coli *model, while 133 where defined as reversible.

As the assignment depends on the estimated Gibbs energies of formation, we performed a sensitivity analysis to assess the assignment's reliablility. We widened the allowed ranges of Gibbs energies of reaction by 1, 2, 3, 4 kJ/mol and performed assignment runs using these. Despite the broadened ranges, 40 out of 43 of our direction assignments based on thermodynamic facts were still valid. Only up to three reactions (depending on the uncertainty range used) would not had been defined as irreversible. As our earlier direction assignments are in-line with the reaction directions in the original model and also in KEGG, we believe that our irreversibility assignments are correct.

### Thermodynamic heuristics-based assignment

The limited availability of experimental Gibbs energies of formation only allowed us to analyze a rather small subset of reactions. Thus, we expanded the direction assignment procedure by another approach. As shown in [20] the reaction network comprises sets of reactions whose simultaneous operation would contradict fundamental thermodynamic principles. Thus, also we aimed at identifying thermodynamically infeasible subnetworks from the metabolic network. In contrast to [20], we used a different kind of subnetwork which will be outlined below. After having identified these subnetworks, heuristic rules were employed to pinpoint the reaction(s) in the identified subnetworks which most likely are irreversible and reaction directions were set accordingly. We employed the co-substrate converting cycles to identify reactions that most likely are irreversible under all conceivable environmental conditions. The direction assignment based on topology and heurisitics was also implemented in the algorithm (cf. Fig. 1, steps 2a–2c).

Note that it is conceivable that a direction assignment based on the topological considerations contradicts an assignment made with the thermodynamic facts. Here, this was, however, never the case. For some reactions the thermodynamics facts were only less restricting as they allowed both directions while a heuristic rule constrained the reaction into one direction. To prevent the exclusion of actually possible reaction directions, we adopted the restriction only if the heuristics-based assignment was highly reliable (see below).

#### Identification of thermodynamically infeasible operation of reaction sets

First, we had to identify sets of reactions (subnetworks) whose simultaneous operation is thermodynamically infeasible. A thermodynamically infeasible operation of a subnetwork is, for example, given by a cyclic operation of a reaction set that in total results in no net conversion of metabolites. The absence of such reaction cycles is a necessary condition for thermodynamically consistent operation of reaction networks [24]. Hence, cycles in the metabolic network are a promising target to screen for thermodynamically infeasible reaction directions. Cycles can be obtained via the null space of the stoichiometric matrix.

**Figure 2:**
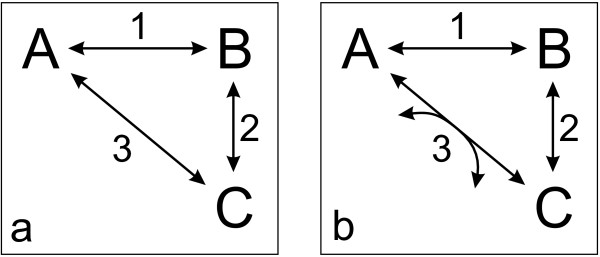
Illustration of reaction cycles.

Consider a network that consists of three reactions with the pairwise interconversion of the reactants A, B and C (cf. Fig. 2a). Assume a situation where A is actually converted to B, and B to C. Thus, C must have a lower Gibbs energy of formation than A. Consequently, the operation of the reaction 3 from C to A is not possible. This example shows that, if we preset a consecutive operation of two reactions, it is possible to exclude one direction of the third. Thus, here, we only can state if-then relationships for reaction directions, and consequently, an *a priori *determination of reaction directions – without the assumption of other reaction directions – is not possible.

Next, we extent the thought experiment and assume a reaction between A and C with a different stoichiometry that is actually able to re-cycle C to A (cf. Fig. 2b). This reaction would have to be driven by a "motor" that delivers the energy necessary to convert the reactant C to the higher energy state of A. In metabolism, chemical energy can be delivered by the conversion of a highly energetic co-substrate (e.g. ATP) to its low energetic counterpart (e.g. ADP). In this case, a cyclic operation of the reactions from A to B, B to C and C back to A is thermodynamically feasible as the system is supplied with energy. On the contrary, the reverse operation of this reaction cycle is thermodynamically infeasible as the "motor" would then operate in opposite direction and would become a "generator": The cycle would produce energy (e.g. in the form of ATP). In order to exclude such thermodynamically infeasible energy production, one of the reactions in this cycle is set irreversible such that the highly energetic co-substrate cannot be produced. The reaction that produces the highly energetic co-substrate was here the prefered target to assign a direction that only allowed energy consumption. In the context of our work, we call a model "thermodynamically infeasible" if no generation of highly energetic co-substrates by a cyclic operation of metabolic reactions is possible.

Several pairs of low/highly energetic co-substrates exist (cf. Table 1). These are pairs of (i) nucleotide phosphates, of (ii) nicotinamide adenine dinucleotides, of (iii) nicotinamide adenine dinucleotide phosphates and of (iv) flavin adenine dinucleotides, and (v) intra- and extracellular protons. Due to the proton motive force over the membrane, extracellular protons are high-energy counterparts to intracellular protons.

**Table 1:** Co-substrate groups that were eliminated from the stoichiometric matrix to identify energy producing cycles

selected co-substrate groups
NTP, NDP, NMP
NADH, NAD^+^
NADPH, NADP^+^
FADH_2_, FAD^+^
Hextracellular+ MathType@MTEF@5@5@+=feaafiart1ev1aaatCvAUfKttLearuWrP9MDH5MBPbIqV92AaeXatLxBI9gBaebbnrfifHhDYfgasaacH8akY=wiFfYdH8Gipec8Eeeu0xXdbba9frFj0=OqFfea0dXdd9vqai=hGuQ8kuc9pgc9s8qqaq=dirpe0xb9q8qiLsFr0=vr0=vr0dc8meaabaqaciaacaGaaeqabaqabeGadaaakeaacqqGibasdaqhaaWcbaGaemyzauMaemiEaGNaemiDaqNaemOCaiNaemyyaeMaem4yamMaemyzauMaemiBaWMaemiBaWMaemyDauNaemiBaWMaemyyaeMaemOCaihabaGaey4kaScaaaaa@40B7@, H^+^

Besides these co-substrates, also the molecules water, oxygen, carbon dioxide, ammonium and inorganic phosphate were removed from the stoichiometric matrix to identify energy producing cycles. This is necessary to elementally balance the resulting net conversion of co-substrates. NTP, NDP and NMP denote nucleoside tri-, di- and monophosphate for adenosine, cytidine, guanosine, inosine and uridine.

To identify cycles that interconvert these co-substrates, again the null space of the stoichiometric matrix was calculated, however, only after having removed the co-substrates' stoichiometric coefficients from the matrix. The respectively obtained null space then included two sets of cycles: (i) the cycles, that do not produce or consume any metabolite, and which were already described by the null space of the original stoichiometric matrix and (ii) cycles that – when complemented with the removed co-substrates – interconvert these. In terms of the terminology introduced in network-based metabolic pathway analysis [25], these two sets of cycles correspond to the extreme pathways of Type III and Type II, respectively. Having complemented the cycles with the co-substrates, we determined the net conversion of co-substrates for each cycle and identified the cycles that convert low energetic co-substrates to their highly energetic counterparts. In the following steps, we worked with this set of cycles to assign reaction directions, and here, we will call these energy producing subnetworks solely 'cycles'.

Every possible energy producing cycle is a combination of the linearly independent basis vectors of the null space of the reduced stoichiometric matrix. As the running time for the computation of all linear combinations increases exponentially with system size [24,26], an exhaustive analysis of all possible cycles is currently not feasible (M. Terzer and J. Stelling, *personal communication*). For this reason, we based our assignment procedure on the cycles that are described by the basis vectors of the calculated null space matrix. As we will see below this approach was not fully comprehensive but allowed excluding thermodynamically infeasible cycling to a large extent while still being computationally reasonable.

Faced with the fact that we only obtained one possible set of linear independent basis vectors, the choice of the null space matrix calculation, however, was important for the assignment procedure. In preliminary tests, when we applied a null space matrix that included larger cycles, our algorithm assigned less reaction directions. Thus, one should apply a null space matrix with cycles that consist of the smallest possible number of reactions. Here, the null space matrix was calculated from the reduced echelon form of the stoichiometric matrix by the Matlab function *null*. The null space of the co-substrate reduced stoichiometric matrix was described by 227 linear independent reaction cycles with an average number of reactions of 8.85 and a median number of reactions of 4. Of all cycles within the null space matrix, 145 were energy producing cycles.

**Figure 3:**
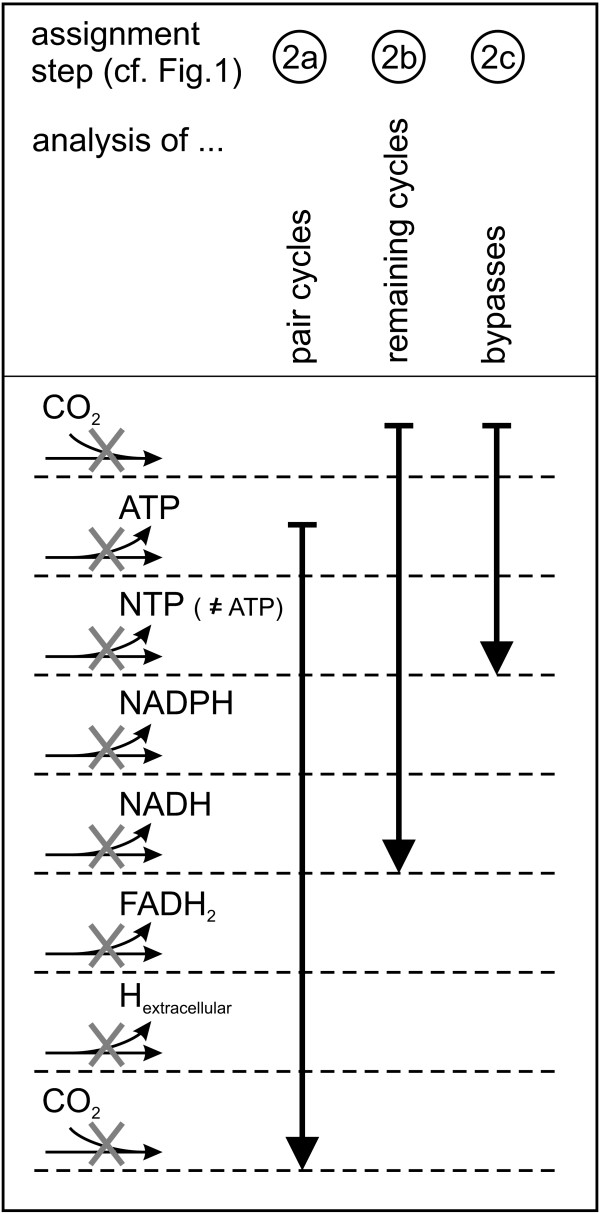
**Illustration of the procedure to assign reaction directions by heuristic rules**. For the assignment steps 2a–2c (cf. Fig. 1), the applied heuristic rules are displayed. Generally, the rules defined a reaction as irreversible in the direction of consumption of a high-energy co-substrate. The rules, however, were not applied if the respective reaction simultaneously produced CO_2_. The vertical arrows indicate the consecutive application of the rules: if no assignment was possible with a particular heuristic rule, the next rule along the arrow was employed. The consumption of a co-substrate with a higher energetic content was prefered over the consumption of a co-substrate with a lower energetic content. In case the cycle contains more than one reaction producing the same highly energetic co-substrate, all these reaction steps are defined as irreversible. In pair cycles (2a), the reaction that produces the only generated co-substrate was defined as irreversible as long as it did not consume CO_2_. In case of CO_2 _consumption, however, it follows that the other reaction also produces CO_2 _and we define this reaction direction as irreversible. As only one co-substrate pair is converted in each pair cycle, the assignment was achieved by applying the heuristic rules consecutively while omitting the first rule as indicated in the figure. In the analysis of the remaining energy producing cycles (2b), the cycles can contain reaction steps that produce different kinds of co-substrates. Here, in the first place we restricted CO_2 _consumption, which is in general indicating a thermodynamically infeasible reaction step. If no CO_2 _consuming reaction was preset, the production of highly energetic co-substrates were disabled with the indicated priorities. Note that NADPH and NADH producing reactions, here, were assigned with the same priority (not illustrated in the figure). In the bypass analysis (2c), reaction directions were assigned for CO_2 _consuming or nucleotide triphosphates producing reactions. Preliminary studies showed that only these heuristic rules were fully reliable in this assignment step, and thus, we only applied these two rules.

This set of cycles was now employed to assign reaction directions by thermodynamics-based heuristic rules: In three steps that are described in the following paragraphs different kinds of cycles were analyzed and reaction directions were assigned by the heuristic rules in Fig. 3. These rules selected for and disabled reaction steps that produce high-energy from low-energy co-substrates. Such, we could assign directions for reactions beyond the ones, for which the Gibbs energies of reaction were available.

#### Analysis of pair cycles

Cycles that consist of only two reactions occur frequently in metabolic networks. Here, the null space matrix contained 45 energy producing pair cycles. For such cycles, the direction assignment to eliminate thermodynamically infeasible energy production is straightforward due to the limited possibilities for assignment of reaction directions: There are only two reactions which can be set irreversible, and the most natural approach is to block the reaction step that produces the highly energetic co-substrate. This was the only heuristics-based assignment step that was allowed to be more restricting than the thermodynamic facts assignment step. Technically, this procedure was realized by applying heuristic rules as explained in Fig. 3.

Applying these heuristics to the identified energy producing pair cycles, 42 reactions were restricted to one direction. As none of these reactions was already previously defined as irreversible in the thermodynamic facts-based assignment, in summary 85 direction assignments were made until here.

#### Analysis of remaining energy producing cycles

The following assignment step (2b in Fig. 1) aimed at defining reaction directions in the remaining energy producing cycles. As these consist of more than two reactions, several conceivable options to disable energy producing cycling typically exist. Hence, it is important to note that this step of the heuristics-based assignment is less reliable.

Applying heuristic rules as depicted in Fig. 3 to the remaining 45 energy producing cycles in the null space matrix, 26 reactions were suggested to be irreversible. Five of these, however, were identified to be reversible in the thermodynamic facts-based assignment. In these cases, we prefered to follow the thermodynamic facts-based assignment for the following reasons: First, the Gibbs energy of reaction is the hard physical ground for a reaction's directionality. Second, by setting a reaction reversible we do not exclude directions that indeed are possible under some physiological conditions. Therefore, only the 21 directions that do not further constrain the thermodynamic facts-based assignments were adopted, and as a result, at this point 106 reaction directions were defined in total.

#### Analysis of bypasses

So far, only the energy producing cycles of the initially calculated null space matrix were analyzed and blocked by the outlined procedure in case the heuristic rules were applicable. As the calculation of all possible cycles is currently not feasible, in the next step (2c in Fig. 1), we at least investigated pairwise combinations of the complete set of available cycles – including also the non-energy producing cycles: In case a second cycle could act as a bypass for an already identified infeasible reaction step of a first cycle, we aimed to exclude the operation of the bypass.

The bypasses were identified as follows. Each reaction, which was defined as irreversible and disabled an energy producing cycle, was analyzed. Among all cycles in null space matrix (also including non-energy producing cycles), we selected for those in which the analyzed reaction occurs. This subset of cycles is capable to bridge the particular reaction of the first cycle, i.e. to form bypasses that start at the reaction's educts and ends at its products. In consequence, the initial cycle, whose thermodynamically infeasible operation was already disabled, and the bypass build a new – potentially energy producing – cycle. For each identified bypass, we first checked whether it was an actually functional bypass given the previously made direction assignments. If the bypass was already blocked, there was no need for any action. Otherwise, we checked whether the co-substrate conversion of the resulting new cycle was thermodynamically infeasible by calculating the cycle's Gibbs energy of reaction. If it was infeasible, a reaction direction within the bypass was assigned by applying the heuristic rules illustrated in Fig. 3.

Analyzing the bypasses that bridge the 106 previously assigned reactions, in a first iteration step 24 additional irreversible reaction directions were defined by the heuristic rules. In a second iteration step, in which we analyzed bypasses for the reactions that were defined as irreversible in the first iteration step, no further directions could be assigned: The bypasses were either already inibited or no further reaction directions could be identified with the employed heuristics.

## Discussion

### Achieved direction assignment

Table 2 summarizes all assignments that were made by our systematic procedure. While the thermodynamic facts-based assignment yielded 43 irreversible reactions, 87 further reaction directions were assigned based on network topology and thermodynamic heuristics. Altogether, 130 reactions were restricted to one direction, which disabled the operation of 129 of the 145 energy producing cycles present in the employed null space matrix.

**Table 2:** Overview over the number of direction assignments made in each step

assignment step	analysis of ...	number of assigned directions	
		in the respective step	in total
thermodynamic facts		43	43

thermodynamic heuristics	pair cycles	42	85
	remaining energy producing cycles	21	106
	bypasses	24	130

Our algorithm did not completely disable thermodynamically infeasible energy production: The heuristics failed in blocking all energy producing cycles and the bypass analysis was not able to identify all possible energy producing cycles. In order to assess the completeness achieved with our approach, we estimated how many additional direction assignments had to be made to completely prohibit infeasible co-substrate conversion. For this, an iterative procedure was applied: A possible energy producing cycle was identified using flux balance analysis, and then, reaction directions were assigned manually to block this cycle (cf. Methods section). When no further energy producing cycles were found, the reactions' directionalities were assumed to reflect thermodynamic feasibility with respect to energy generation. At this point, the direction assignment was considered to be complete.

With this procedure, 59 additional assignments of reaction directions were required until infeasible energy production was excluded. Simulating aerobic growth on glucose by flux balance analysis, ATP was then produced via the respiratory chain. Importantly, the production of energy equivalents such as ATP by metabolic reactions was not generally rendered impossible by our linear constraints as our algorithm only selectively disables the generation of highly energetic co-substrates. In summary, the 189 irreversible reactions (of which 130 were assigned by our algorithm) were sufficient to yield a thermodynamically reasonable model with respect to the production of energy equivalents.

At this point, we checked whether the application of general biochemical rules such as defining all kinase reactions as irreversible would have been a much simpler and also valid alternative to our approach. A close inspection of the 74 kinase reactions in the model revealed that this would not had resulted in a correct model: For instance, the phosphoglycerate kinase reaction is known to operate in both directions and it is correctly defined as reversible in our assignment. This demonstrates that employing heuristic rules in combination with analyzing co-substrate converting cycles is superior to simple general biochemical rules.

With the model analyzed here, the calculation time required for the assignment procedure was roughly two minutes on a Pentium 3 GHz PC, if the calculation of the null space matrix and generation of a Excel file for output documentation is included. The assignment algorithm itself required about 30 to 40 s. Such, the computational effort is small and the algorithm can be efficiently executed on a usual PC.

### Comparison to original model

The introduced systematic direction assignment yielded 130 reactions that were restricted in one direction. Together with the 59 manual assignments that eventually eliminated any thermodynamically infeasible cycling, we obtained 189 reactions that are irreversible in our model. In comparison to the 676 irreversible reactions in the original model from Palsson and co-workers [4], this is a rather small number and indicates a much less constrained model.

From a constraint-based modeling viewpoint, a direct comparison of the number of irreversible reactions, however, is misleading as one assigned reaction direction can practically render impossible the reversible operation for a set of other reactions. For example, one irreversible reaction that is part of an unbranched linear pathway restricts the operation of the whole pathway to one direction. Hence, in effect it is no difference if the direction of only one or all reactions of the pathway are defined as irreversible.

To allow for assessment of model flexibility due to different direction assignments, we had to identify correlated sets of reactions (cf. Methods). Using the identified correlated sets, the number of *de facto *irreversible reactions was assessed. We found that the stoichiometric network of *E. coli *comprises 175 sets of correlated reactions. If one reaction in such a set is defined as irreversible, mass balance constraints rule out one particular direction for each of the other reactions in the set. In the original model, 749 reactions are practically irreversible. In comparison, our direction assignment eventually resulted in 292 reactions that practically can operate only in one direction.

We found that only in one case – namely the UTP-glucose-1-phosphate uridylyltransferase reaction – our algorithm defines a reaction as irreversible which is reversible in the original model. Remarkably, our assignment is in agreement with the EcoCyc database [27] which also states that this reaction is irreversible.

As the predicted maximal biomass yield on glucose is increased by about 20% using our reaction directions in comparison to the original, the model with our reaction directions is much less constrained and there are more possibilities to distribute the mass flux through the reaction network. Therefore, it is envisioned that it covers a larger range of metabolic scenarios, e.g. knockout mutants or different environmental conditions. As an example, a *frdA *deletion mutant (*in vivo *viable when grown anaerobically on glucose [28]) is *in silico *nonviable with the original reaction directions while it is viable with our reaction directions.

### Extension of heuristic rules

Next, we evaluated whether we could complement the employed heuristic assignment rules to increase the number of reactions that are automatically defined as irreversible. Additional or modified heuristic rules should eliminate the energy producing cycles that were not yet disabled by our algorithm.

First, we closely inspected the additional manual direction assigments that were required to eliminate all the remaining energy producing cycles (cf. Additional file 1). In this reaction set, we found reactions, which potentially could have been made irreversible by the heuristics already used in the algorithm, i.e. reactions that produce/consume high-energy/low-energy co-substrates, but for several reasons (as outlined above), the respective directions were not assigned. There are, however, groups of reactions (e.g. quinone pool reducing/oxidizing reactions) whose common attributes could be exploited by new heuristics that specifically assign directions to such sets of reactions (cf. Table 3).

**Table 3:** Number of additional direction assignments required to eliminate remaining thermodynamically infeasible energy production

common attributes	standard procedure	standard procedure with consideration of final electron acceptors
quinone pool reductions	15	-
transporters	9	8
NTP production	14	12
NADH/NADPH production	5	4
O_2 _production	2	1
CO_2 _consumption	4	4
NMP synthesis	7	7
other	3	3

sum	59	39

**Figure 4:**
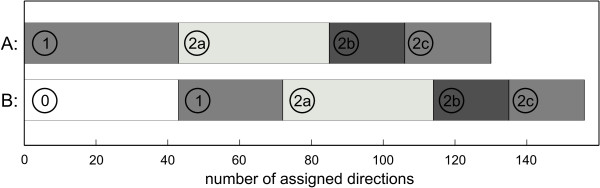
**Comparison of the assignment where final electron acceptors are considered to the default assignment**. The numbers of made direction assignments of the standard assignment procedure (A) and the assignment procedure, which additionally drew on the direction of electron transfer within the respiratory chain (B) are compared. The numbers (1 – 2c) refer to the assignment steps depicted in Fig. 1, while step 0 represents the reaction directions that were assigned by the additional heuristic rule based on final electron acceptors.

As an example for such an extension of the heuristic rules, the quinone pool converting reactions were set as irreversible such that the electrons are transferred from the reduced metabolites to the final electron acceptors. Having defined the final electron acceptors, it was possible to assign 43 reaction directions in the *E. coli *model. When we incorporated this rule into the assignment algorithm, in total 26 more reactions were restricted in one direction (cf. Fig. 4). Fourteen out of the 43 reactions had been already assigned by the thermodynamic facts, and the bypass analysis assigned three reactions less. In summary, 156 instead of 130 reactions could then be defined as irreversible by our systematic assignment procedure.

The extension of heuristic rules by organism-specific knowledge obviously is an effective and effortless approach to increase the number of assigned directions. Similarily, one could define the directions of the transporters according to their function, which often can be identified from stoichiometry alone (e.g. sugars are taken up by PTS systems).

## Conclusion

This paper reports on a computational framework that – based on thermodynamic principles – systematically assigns reaction directionalities in genome-scale stoichiometric metabolic models. We demonstrated its application on a metabolic reconstruction of *E. coli*. After having exploited all available thermodynamic data to define irreversible reactions, we drew on network topology and thermodynamic heuristics to assign further reaction directions: Energy producing cycles were extracted from the reaction network and thermodynamically infeasible reaction steps that produce high-energy from low-energy co-substrates were disabled.

The proposed direction assignment procedure has several advantages over other approaches. The group contribution method to computationally estimate the Gibbs energies of formation is associated with such large uncertainties that only five reactions could be identified as irreversible in a genome-scale model [18]. The method developed by Beard and co-workers for *ab initio *prediction of reaction directions [20] relies on the availability of all possible cycles in the metabolic network. Currently, these cannot be calculated with reasonable computational effort for genome-scale models and the method also does not completely disable thermodynamically infeasible cycling. In contrast, using our algorithm, we demonstrated that a large number of assignments could be made without laborious calculations: A total of 130 directions could be assigned automatically, which constitutes a large fraction of the direction assignments necessary to exclude thermodynamically infeasible energy production.

Along with the development of mathematical methods that employ genome-scale metabolic models, these models became valuable tools in systems biology and metabolic engineering. Here, our systematic assignment procedure can be used in the reconstruction of new models or in the revision of existing ones. Currently, large efforts are put into the automated reconstruction of such models [10,29] and several computational tools exist that support the first steps of the reconstruction process [11,30]. On the contrary, the following steps towards finalizing the model – which include the definition of reaction directionalities – are still done manually. We envision that the here proposed algorithm could be a valuable part of a computational framework that assists the automated reconstruction process for genome-scale metabolic models.

## Methods

### Employed software package

All calculations were carried out employing Matlab (The MathWorks Inc., MI, USA) unless specified otherwise. Necessary input data are standard Gibbs energies of formation and physiological ranges of intracellular metabolite concentrations. As output, the algorithm generates a vector which specifies the assigned reaction directions, and in addition, creates a detailed report (in Microsoft Excel) on the respectively made assignments.

### Applied metabolic network model

For the *E. coli *data set, we employed the genome-scale model iJR904 [4]. This model is an elementally balanced stoichiometric network and such enabled the calculation of the reactions' Gibbs energies. The model was slightly modified by eliminating one reaction of duplicate reaction pairs, i.e. reactions that occur twice in the original model's list of reactions. Moreover, the artificial reaction that accounts for the cell's maintenance requirements in the model was omitted. The model is supplied in Additional file 1.

### Employed Gibbs energies of formation and concentration ranges

A prerequisite for the thermodynamic facts based direction assignment is the availability of standard Gibbs energies of formation for a large number of metabolites. With these and values for intracellular pH and ionic strength (see below), standard transformed Gibbs energies of formation specific for intracellular conditions were calculated using the software Mathematica (Wolfram Research Inc., IL, USA) and a Mathematica notebook provided on [31]. Standard transformed Gibbs energies of formation for the metabolites involved in the pentose phosphate pathway and the shikimate pathway were added by drawing on data from the NIST database on thermodynamics of enzyme-catalyzed reactions [32] and from the literature [33,34]. For the Gibbs energies of formation of the quinones in the model, the values of reduced and oxidized ubiquinone, which is the only quinone available in the database, were assumed respectively. Transformed Gibbs energies of formation were adjusted to *E. coli*'s intracellular pH of 7.6 [35] and ionic strength of 0.15 M [36] (cf. Additional file 2).

To reflect typical cytosolic concentrations, which lie in the *μM *to *mM *range [23], the intracellular concentrations' upper and lower bounds were by default set to 0.001 *mM *and 10 *mM*, respectively. Exceptions were made for oxygen, for which the upper limit was set to 0.1 *mM *to account for its low solubility, and carbon dioxide and inorganic phosphate, for which ranges from 1 to 50 *mM *were assumed.

### Manual elimination of energy producing cycles

The iterative and manual direction assignment to eliminate all remaining energy producing cycles was carried out as follows: To detect a thermodynamically infeasible cycling, a flux distribution was generated by means of flux balance analysis using maximal growth rate as optimization objective (cf. [37]). Shortly, such calculated flux distributions comprise the rate of each reaction such that (i) the conversion of each metabolite is balanced, and (ii) glucose (as the employed carbon source) is converted to as much biomass as possible. As the production of biomass requires energy, a part of the glucose has to be metabolized to CO_2 _to yield the necessary chemical energy. Energy producing cycles render the investment of glucose into energy dispensable, and essentially all glucose is converted to biomass. In this case, the calculated flux distribution comprises at least one thermodynamically infeasible energy producing cycle, and can be used to identify the reactions that make up this cycle.

Having identified these cycles, we manually defined directions for one or more reaction in the set of reactions such that the identified infeasible cycling is disabled. The employed rationales for the assignment were similar to the heuristics used in the presented algorithm. Essentially, reactions that consume low-energy or produce high-energy co-subtrates were selected. In some cases, this was not possible as also low-energy metabolites were produced or high-energy metabolites were consumed concomitantly. Then, we determined reaction directions according to the metabolic function of the respective enzyme.

### Calculation of sets of correlated reactions

Two reactions are correlated if the ratio of their reaction rates is identical under any conceivable condition. To identify sets of correlated reactions, in a first step, the stoichiometric matrix was extended by exchange reactions and a reaction describing biomass formation to determine mass balancing sets of reaction rates, i.e. flux distributions. Here, exchange reactions were coupled to all extracellular metabolites and enabled their interchange with the environment. Next, we calculated the null space matrix for this extended stoichiometric matrix. Rows of this null space matrix that are linearly dependent indicate that the corresponding reactions are correlated. Sets of correlated reactions were determined by an all-against-all comparison of the rows.

## Authors' contributions

AK, SP and MH designed the research. AK developed the algorithm. AK and MH analyzed the results. All authors read and approved the final manuscript.

## Supplementary Material

Additional file 1 Lists of assigned reaction directions. This Excel file contains three sheets with (i) the list of reactions that were assigned by the standard assignment procedure, (ii) the list of reactions in case the final electron acceptors are additionally considered, and (iii) the lists of reactions that were assigned manually to obtain a thermodynamically reasonable model. In the first two sheets, we also report in which assignment step a direction was defined by our algorithm. Click here for file

Additional file 2 List of applied Gibbs energies of formation. This Excel file contains the list of the model's metabolites and – if available – the respective transformed Gibbs energy of formation at a pH of 7.6 and an ionic strength of 0.15 *M*. Click here for file
